# Initial Validation of the Diabetes and Breastfeeding Management Questionnaire (DBM-Q)

**DOI:** 10.3390/ijerph17093044

**Published:** 2020-04-27

**Authors:** Karolina Linden, Marie Berg, Carina Sparud-Lundin, Annsofie Adolfsson, Jeanette Melin

**Affiliations:** 1 Centre for Person-Centred Care, Institute of Health and Care Sciences, Sahlgrenska Academy, University of Gothenburg, 405 30 Gothenburg, Sweden; marie.berg@fhs.gu.se (M.B.); carina.s-lundin@fhs.gu.se (C.S.-L.); 2 Department of Obstetrics and Gynecology, Sahlgrenska University Hospital, 416 50 Gothenburg, Sweden; 3 School of Health Sciences, Örebro University, 701 82 Örebro, Sweden; annsofie.adolfsson@oru.se; 4 RISE Research Institutes of Sweden, 412 58 Gothenburg, Sweden; jeanette.melin@ri.se

**Keywords:** Rasch Measurement Theory, breastfeeding, diabetes management, postpartum, diabetes mellitus

## Abstract

Women with pre-gestational diabetes face additional challenges after birth as they struggle with breastfeeding and managing unpredictable blood glucose levels. The aim of this study is to validate the Diabetes and Breastfeeding Management Questionnaire (DBM-Q). In total, 142 mothers with type 1 diabetes mellitus answered the questionnaire, which initially consisted of 11 items. The response rate was 82.5% (*n* = 128) at two months, and 88.4% (*n* = 137) at six months postpartum. The measurement properties of the Diabetes and Breastfeeding Management Questionnaire were tested according to the Rasch measurement theory (RMT). One item showed both disordered thresholds and several model misfits and was removed. Two items showed disordered thresholds which were resolved by collapsing response categories. This resulted in a 10-item questionnaire with all the fit residuals within the range of +2.5, minor significant differential item functioning, well-targeted items and a person separation index of 0.73. Evaluating the DBM-Q according to the RMT is a strength, as it evaluates data against strict measurement criteria. This study provides an initial validation of the questionnaire. The DBM-Q shows good measurement properties for measuring diabetes and breastfeeding management postpartum in women with pre-gestational diabetes. Further studies are needed to identify cutoffs for when professional support is needed.

## 1. Introduction

Person-centered outcome measures with self-reported questionnaires are central to capturing patients’ health status and their own perspectives on received healthcare [1]. This facilitates personally tailored care and provides healthcare professionals with insights into areas for improvement, enabling them to identify, prioritize and make strategic decisions in these areas at an organizational level [2]. The retrieval of valid person-centered outcome measures and measurement properties needs to be evaluated [3].

Women with pre-gestational diabetes mellitus face additional challenges and are more exposed in the first period after birth, as they often have to struggle with breastfeeding and managing unpredictable blood glucose levels [4,5,6,7,8]. Although the rate of breastfeeding in mothers with diabetes mellitus varies worldwide, there appears to be a trend that breastfeeding initiation is lower in this group compared with mothers not affected by diabetes [9,10,11,12,13,14]. Research indicates that women with pre-gestational diabetes mellitus who express a strong intention to breastfeed pre-birth are more likely to continue to do so at three months postpartum [15]. However, even if the will to breastfeed is strong, hurdles faced along the way may affect their ability to initiate and maintain successful breastfeeding. This struggle may be even more difficult for women in rural settings who may find it harder to access specialized health care. During pregnancy, women outside of cities are more likely to receive care in a disconnected diabetes organization, where they are forced to act as messengers between different care providers [16]. After childbirth, the professional support for the mother often ends abruptly as the health services’ focus is shifted towards the newborn rather than the mother’s health. This shift may prompt feelings in new mothers of being disconnected from diabetes-related health care [4]. Typically, in Sweden, a woman visits her midwife between eight and twelve weeks postpartum, and the family receives one home visit from a pediatric nurse who may help with breastfeeding concerns. About half of the women will visit with their regular diabetes care team within two months, and almost all at six months after childbirth [6]. Being in a rural setting may affect the accessibility of services. If a woman lives in the city, she may rent a breast pump and have access to a specialized lactation specialist more easily than a woman living more remotely. Some resources, but far from all, are offered in a digital setting. As part of a research project of women with type 1 diabetes mellitus, a set of questions was developed to capture the situation of being a “breastfeeding mother with diabetes”. Thus, it was considered necessary to include questions related to both breastfeeding and diabetes management issues, and at the same time evaluate the questionnaire according to state-of-the-art principles for measurement properties.

Due to the unique challenges that women with pre-gestational diabetes face postpartum, there appears to be a lack of validated instruments that measure their situation as breastfeeding mothers. For example, the breastfeeding self-efficacy scale [17] is clinically useful for identifying women in need of professional support with lactation. It does not, however, capture the exceptional situations that emerge from having to manage diabetes while breastfeeding. The PostTrans Questionnaire [18] was developed simultaneously to our questionnaire. It assesses psychosocial well-being in women with pre-gestational diabetes while transitioning to motherhood. The questionnaire consists of 27 items and measures six factors. Although breastfeeding is assessed in the questionnaire, it is not the focus, while our questionnaire aims to explore the ability to manage during this phase. In this paper, we present a validation of the Diabetes and Breastfeeding Management Questionnaire (DBM-Q).

## 2. Materials and Methods

In order to fulfil its aim, an instrumental design study was conducted utilizing the Rasch measurement theory (RMT). The DBM-Q was developed by the MODIAB research group based on clinical experiences, literature reviews and focus group discussions with the target population. Previous qualitative research indicated that breastfeeding impacts on daily structure and on routines related to diabetes management [4]. Hence, the topics covered were diabetes management, including glycaemic control, breastfeeding, and support in early motherhood. To assure face validity, 20 women tested the DBM-Q for comprehensibility and relevance, leading to minor revisions. A first version of the DBM-Q was used in a study consisting of 108 women with type 1 diabetes mellitus, two and six months postpartum [8]. The data used in this validation come from a second version of the questionnaire consisting of 11 items [6].

### 2.1. Participants and Data Collection

The data for this study were obtained from a previous randomized controlled trial (RCT) conducted by the MODIAB research group between November 2011 and December 2014. The aim of the RCT was to evaluate web-based support, including reliable information for pregnant women and new mothers with type 1 diabetes mellitus in Sweden [19]. The questionnaire was mailed out and completed at home. The study participants consisted of 155 pregnant women with type 1 diabetes mellitus who were registered at any of the six participating hospital-based antenatal care centers situated in the western and central parts of Sweden, in both urban and rural settings. Details on the data collection were reported elsewhere [19,20]. Of the 155 participating women, 142 women answered the questionnaire on at least one occasion. The response rate was 82.5% (*n* = 128) at two months, and 88.4% (*n* = 137) at six months postpartum. The demographic data are presented in Table 1.

### 2.2. Ethical Considerations

Ethical approval for the original study, from which the data were obtained, was attained from the Ethics Committee of Gothenburg, Sweden (No. 659-09). All the participants in the original study (from which the data were derived) signed an informed consent form after they had obtained written and oral information about the study.

### 2.3. Measurement

During the pre-testing, the DBM-Q consisted of eleven items in total, presented in Table 2. One item with the response options: positive, fairly positive, fairly negative, and negative; one item with the response options: very important, important, fairly important, and unimportant; two items with the response options: to a great extent, to some extent, somewhat, and not at all: four items with the response options: to a high degree, to some extent, somewhat, and not at all; one item with the response options: I got the support I needed, I needed more support, and I received too little support; one item with the response options: much more than usual, somewhat, and not at all; and finally, one item with the response options; yes, partly, and no. In the analyses, four items (AMN8, AMN9, AMN12 and DIA19) were reversed, so a higher raw score indicated higher diabetes and breastfeeding management.

### 2.4. Rasch Measurment Theory (RMT)

With the intention of having invariant measures, the Danish mathematician Georg Rasch developed a model, the Rasch measurement theory (RMT), based on the same underlying principles as for physical measurements [21]. Briefly, the RMT estimates person- and item-attribute values separately and provides scaling on a common interval logit scale. In turn, this enables more accurate measurements that are measured independently of the sample [3,21,22].

Initially, the RMT model focused on dichotomous (e.g., yes/no or pass/fail) items as a logistic function of the relative difference between the respondents’ and items’ locations at the same continuum [21]. It is based on how measurements can be derived through postulates that the odds of a “yes or pass” response corresponds to the probability of a “yes or pass” response, divided by the probability of a “no or fail” response. This provides separate measures for the respondent and the item on the same interval scale, corresponding to the measurement continuum. This dichotomous model was subsequently expanded to a polytomous model (i.e., Likert scales) [23].

Cano and Hobart [24] summarized the major criticism against the classical test theory (CTT) as: (i) ordered counts are not interval measures, (ii) results for scales are sample dependent, (iii) results for samples are scale dependent, (iv) missing data cannot be handled directly, (v) there is a lack of scaling items and (vi) the error around an individual person’s score is a constant value, regardless of the person’s location on the measurement continuum. However, this is overcome by the RMT, which ensures invariant measurements [24].

### 2.5. Statistical Analysis

The measurement properties of DBM-Q were tested according to the RMT by using the software Rasch Unidimensional Measurement model 2030 (RUMM). The analysis focused on the fundamental aspects of the RMT, namely, response category functioning, model fit, differential item functioning, targeting and reliability [25,26].

#### 2.5.1. Response Category Functioning

To evaluate the monotonicity of item response categories, the threshold orders were evaluated. This implies that the ratings for one item should be consistent with the metric estimate of the underlying construct. The collapsing categories were considered when disordered thresholds occurred [25].

#### 2.5.2. Model Fit

The items’ fit residuals, χ^2^, item characteristic curve and local dependency were evaluated for the fit to the Rasch model. (i) Fit residuals, i.e., standardized residuals that summarize the difference between observed and expected responses, should ideally have a mean close to zero (0) and standard deviations (SD) close to 1, and at the same time the individual item fit residuals should ideally be within the range of −2.50 to +2.50. (ii) χ^2^-tests evaluate the difference between the observed and expected item responses and should ideally not be statistically significant (after Bonferroni correction). (iii) The item characteristic curve graphically describes the relationship between the observed and expected item responses, and the dots of the class intervals should ideally follow the item characteristic curve to support good fit [26]. (iv) Local dependency, i.e., whether items are linked, such as responses to one item determining the response to another, were evaluated according to a relative cutoff. This means that the residual correlations >0.20 above the average correlations indicate local dependency [27,28]. In addition, Smith’s method for testing unidimensionality was applied [29]. This means that the patterning of residuals provided from a principal component analysis were examined and two subsets of items were defined in the first residual factor by dividing positively and negatively correlated items. The person estimates for each subset were then compared by using an independent *t*-test, and to support unidimensionality, the percentage of tests outside the range of −1.96 to +1.96 should ideally not exceed 5%.

#### 2.5.3. Differential Item Functioning

Analyses were conducted to evaluate the extent to which item responses are influenced by factors external to the measured construct’s differential item functioning. The item function should ideally be similar across different groups, i.e., non-significant (after Bonferroni correction) in a two-way analysis. Both uniform and nonuniform differential item functioning were tested for these external factors: (i) time of measure: at 2 months and 6 months after giving birth, (ii) insulin administration in early pregnancy, (iii) parity, (iv) mode of birth, (v) gestational age group: <week 30, week 30–38 or >38, and (vi) years with diabetes: 1–9 years, 10–19 years, 20–29 years or >30 years.

#### 2.5.4. Targeting

Ideally, person locations should mirror the item locations, and comparing the mean person location with the mean item location (i.e., 0 logits) indicates whether the sample is off-center of the items [25]. The proportions of extremes at the lower and upper end of the scale were also assessed and interpreted as follows: >5% = poor; 2%–5% = fair; 1%–2% = good; 0.5%–1% = very good; and <0.5% = excellent [30].

#### 2.5.5. Reliability

To evaluate the questionnaire’s ability to separate persons, the reliability was estimated based on the Person Separation Index. It was interpreted as follows: zero (0) indicated all errors, 1 implied no error, >0.70 was required for group assessments and >0.85 was required for individual high-stake evaluation items [25,31].

## 3. Results

Summary item statistics for the DBM-Q (11- and 10-item versions, respectively) are presented in Table 2.

### 3.1. Response Category Functioning

Two items had disordered thresholds. For item AMM9 (How important it had been to breastfeed), it was difficult to differentiate between the response options “not at all” and “somewhat”, and for item DIA21 (If additional support had been received from family members to manage diabetes during breastfeeding) the response “partly” could not be differentiated from “yes” or “no”. Thus, “not at all” and “somewhat” (AMM9) were collapsed, as well as “partly” and “yes” (DIA21). In addition, by studying categorical probability curves, and taking the measurement uncertainties into account for item AMM11 (To what extent had the breastfeeding burdened everyday life), we decided to collapse the response options “not at all” and “somewhat”. Subsequently, item AMN9 still showed disordered thresholds, and it was therefore collapsed into a dichotomous item, i.e., “not at all” and “somewhat”, representing one response option, with “important” and “very important” representing the other response option.

### 3.2. Model Fit

**Figure 1:**
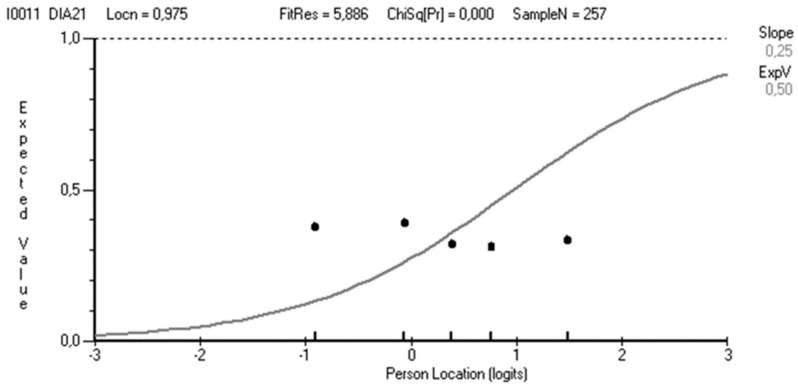
The item characteristic curve for DIA21, showing dots (i.e., observed values) deviating from the line (i.e., expected values) as an indication of misfit.

The item fit residual mean was close to zero, 0.21 (SD 2.47), and most of the item fit residuals were within the range +2.5. As shown in Table 2, item DIA21 showed particularly high fit residual as well as significant χ^2^. The misfit of item DIA21 was also confirmed by studying the item characteristic curve, where the dots deviated from the line (Figure 1). Taking those misfits into account, and the somewhat qualitative differentiating meaning of item DIA21, we decided to remove it. This improved the mean item fit residuals to 0.15 (SD 1.62), and item DIA18 (Problems with low blood sugar during breastfeeding) then showed fit residuals within the range +2.5. Local dependency was a clear problem, with 25 out of 55, and nine out of 45 residual correlations above their respective cutoffs, irrespective of whether DIA21 was included or not. Table 3 shows a correlation matrix of the 10-item version, with emboldened items for higher than the relative cutoff of 0.09. Moreover, the *t*-test for unidimensionality failed at slightly above 15%, both when item DIA21 was included and when it was not.

### 3.3. Differential Item Functioning

There were neither statistically significant differential item functioning main effects, nor interaction effects when the item DIA21 was included, i.e., all items performed similarly for all groups (measured at 2 or 6 months postpartum, insulin administration in early pregnancy, parity, mode of birth, gestational week and years with diabetes). When DIA21 was removed, item DIA20 (Need of support from family members to manage diabetes during breastfeeding) showed a significant uniform differential item functioning for the mode of birth (*p* ≤ 0.01) and the gestational group (*p* ≤ 0.01), although it should be interpreted with some caution as there were only a few women in some of the groups (e.g., VE *n* = 24, and elective caesarean *n* = 49).

### 3.4. Targeting

**Figure 2:**
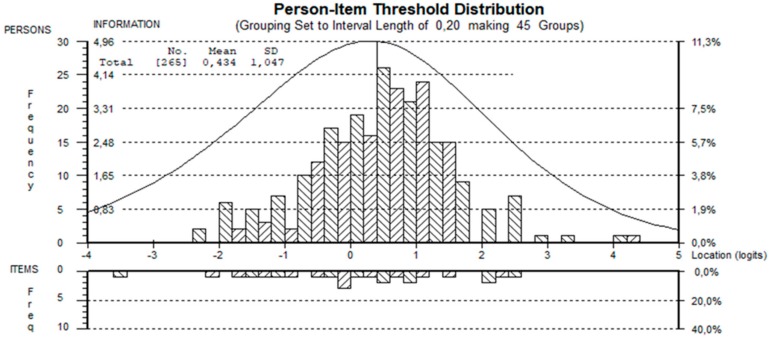
Person-Item threshold histogram for the DBM-Q with 10 items.

As shown in Figure 2, the items’ locations are covered by the persons’ locations and vice versa. The patient mean locations were 0.33 (SD 0.96) and 0.43 (SD 1.05), respectively, when DIA21 was included or not. The proportions of extremes were excellent, i.e., <0.05 independently, both when DIA21 was included or not.

### 3.5. Reliability

The Person Separation Index was 0.73 and 0.74, respectively, when DIA21 was included or not, i.e., the scale’s ability to discriminate between persons’ ability was above the criterion of 0.70 for group evaluations.

## 4. Discussion

This study analyzed a new questionnaire for the assessment of diabetes and breastfeeding management in mothers with type 1 diabetes mellitus. The DBM-Q fills a gap and facilitates the capture of the women’s experiences of diabetes management during breastfeeding and the impact on everyday life. Overall, the DBM-Q shows good measurement properties.

Based on the fit statistics, item DIA21 (Have you received the support you needed from family members in order to manage your diabetes during the breastfeeding period?) is questionable. Its meaning is also somewhat different from the others, as it asks what other people around have done, rather than how the woman herself experienced her situation. Thus, we recommend using a scale of ten items for a higher ordered construct of diabetes and breastfeeding management to be measured. Nevertheless, item DIA21 could be of clinical significance regarding how well mothers are supported at home, especially considering their management of potential unstable glycaemia during breastfeeding initiation. A recent study indicated that the risk of night-time hypoglycaemia in breastfeeding women with type 1 diabetes mellitus might be lower than previously believed [32]. Still, the women may experience fear of such hypoglycaemic episodes, affecting their well-being during this time. We therefore recommend further studies to address the re-formulation of DIA21 and explore opportunities to capture both the need of support, as well as gauge if the women received it to the extent she would have liked. Although item DIA19 (Have you received the support you needed from health care professionals in order to manage your diabetes during the breastfeeding period?) has, to some extent, a dual statement like DIA21, it fits the model. Due to the fit statistics, we retained DIA19 in the DBM-Q, but like DIA21, this item also needs further evaluation.

The DBM-Q was evaluated according to RMT, which is a strength compared to many other questionnaires that build on the classical test theory. An example of a classical test theory based questionnaire is the PostTrans Questionnare, that, similarly to the DBM-Q, focuses on postnatal well-being in the transition to motherhood for mothers with diabetes [18]. However, our study provides several benefits, as data are evaluated against strict measurement criteria rather than described, as with classical test theory [3,26]. The authors of the PostTrans Questionnaire argue for less items to reduce the respondent burden of filling in the questionnaire [18]. In contrast to that, we would argue for more items to be added to the DBM-Q. It might take longer to fill in, but at the same time, the measurement uncertainties will decrease, and consequently, the risk of incorrect and unreliable decisions will decrease. It would be more burdensome to the mothers to not receive the care they need than to fill in some extra items. It is of greatest importance that the women’s health statuses are captured properly to ensure tailored interventions.

In addition to the benefits of applying the RMT, the evaluations are done based on 265 responses from 142 participants, both figures being well-above recommendations for the RMT [33], and a rather high number of women in such a limited population [1]. However, some limitations should be considered when interpreting the findings. Firstly, if only Smith’s *t*-test is relied upon, the unidimensionality is questionable. That said, it could be dangerous to adopt a hardline data-driven approach, as this relies too heavily on the quality of data [1]. On the other hand, qualitative evaluations of a successful ruler [26], as well as supporting fit statistics [25,26] provide evidence for suggesting that a higher ordered construct can be measured. Secondly, local dependency was present for approximately half of the item pairs. This should nevertheless be interpreted with caution as there are only 11 items, and evaluations of local dependency seem to be less reliable when there are fewer than 20 items [27].

Our previous studies have revealed that breastfeeding could negatively influence diabetes management in mothers with type 1 diabetes mellitus. A majority reported considerably more unstable and lower glycaemia, and increased numbers of hypoglycaemic episodes, especially during the first two months. These conditions influence well-being in terms of general health and vitality, i.e., the greater the negative effect on well-being, the more breastfeeding affects diabetes management [8]. Additionally, in a more recent study, we found that a less negative impact of breastfeeding on daily diabetes routines at postpartum correlated with a higher degree of general well-being. A clinically applicable questionnaire that measures these experiences can be used to identify those with more problems during the postpartum period [6]. This is especially important for women living more remotely, who may experience a greater disconnection to diabetes care services postpartum. In both these studies, the DBM-Q was used. A next step will be to determine clinical ‘signal values’, i.e., cutoffs for where the self-reported values correspond to a greater need for professional intervention and support in relation to diabetes and breastfeeding management during the postpartum period. For caregivers of women in rural settings, the completion of the DBM-Q at home may help to identify those in greater need of professional support to maintain successful breastfeeding. With ‘signal values’ in place, it may also be used to select which women need to be prioritized for early intervention, whether in a digital form or as a traditional visit at the clinic.

## 5. Conclusions

The DBM-Q shows good measurement properties for measuring diabetes and breastfeeding management postpartum in women with pre-gestational diabetes, according to the Rasch Measurement Theory. The DBM-Q could therefore be used as a person-centered outcome measure for breastfeeding mothers with diabetes mellitus. Further studies are needed to identify cutoffs for when professional support is needed.

## Figures and Tables

**Table 1:** The demographic data of the participants.

Demographic Data, *n* = 142		Year	Mean	SD	*n*	%
Age			30.98	4.59	142	100
University education					96	68
Primipara					80	56
Insulin Administration						
	Multiple daily injections				90	63
	Pump				52	37
Gestational week at childbirth			37.56	2.12	141	99
Mode of birth						
	Normal vaginal birth				54	38
	Instrumental vaginal birth				12	8
	Emergency caesarean section				8	34
	Elective caesaren section				27	19
Parental leave						
	Full time				121	85
	Part time				2	1
	Not at all				1	1
Breastfeeding						
	Two Months				99	70
	Six Months				70	49

**Table 2:** Summary item statistics of the DBM-Q for versions with 11 and 10 items, respectively.

		11 Items					10 Items				
Item		Location	2SE	Fit residuals	χ2	Probability	Location	2SE	Fit residuals	χ2	Probability
AMM8	What is your experience of breastfeeding?	−1.68	0.22	0.14	4.89	0.30	−1.61	0.22	0.60	3.97	0.41
AMM9	How important is/was breastfeeding to you?	−1.28	0.34	0.32	5.85	0.21	−1.21	0.34	0.60	7.91	0.10
DIA19	Have you received the support you needed from health care professionals in order to manage your diabetes during the breastfeeding period?	−0.80	0.21	1.68	3.62	0.46	−0.75	0.22	1.97	5.65	0.23
AMM12	To what extent has breastfeeding enriched your everyday life?	−0.59	0.17	1.18	2.06	0.72	−0.52	0.18	1.85	8.62	0.07
DIA20	Have you needed support from family members to manage your diabetes during the breastfeeding period?	−0.07	0.20	-0.08	1.74	0.78	0.03	0.21	0.47	2.47	0.65
DIA15	To what degree has breastfeeding had a negative impact on your daily diabetes routines?	0.25	0.17	-0.44	1.99	0.74	0.35	0.18	-0.07	1.74	0.78
AMM11	To what extent has breastfeeding been a burden to your everyday life?	0.51	0.20	1.46	5.30	0.26	0.63	0.20	1.92	4.94	0.29
DIA18	Have you experienced more low blood glucose levels during the breastfeeding period?	0.78	0.16	-2.95	11.75	0.02	0.91	0.16	-2.35	9.93	0.04
DIA16	Have you needed to check your blood glucose levels more often during the breastfeeding period?	0.87	0.16	-2.42	11.50	0.02	1.00	0.16	-1.69	8.16	0.09
DIA17	Have you experienced more unstable blood glucose levels during the breastfeeding period?	1.03	0.17	-2.42	13.32	0.01	1.17	0.17	-1.85	9.61	0.05
DIA21	Have you received the support you needed from family members in order to manage your diabetes during the breastfeeding period?	0.98	0.28	5.89	52.97	0.00					

Bold numbers indicate misfit: Fit residuals should ideally lie between −2.50 and 2.50 and χ2 should not be significant after Bonnferroni correction.

**Table 3:** Residual correlations of the DBM-Q, 10-item version.

Item		AMM11	AMM12	AMM8	AMM9	DIA15	DIA16	DIA17	DIA18	DIA19	DIA20
AMM11	To what extent has breastfeeding been a burden to your everyday life?	1									
AMM12	To what extent has breastfeeding enriched your everyday life?	**0.178**	1								
AMM8	What is your experience of breastfeeding?	**0.327**	0.034	1							
AMM9	How important is/was breastfeeding to you?	**0.42**	**0.316**	**0.157**	1						
DIA15	To what degree has breastfeeding had a negative impact on your daily diabetes routines?	−0.238	−0.207	−0.155	−0.313	1					
DIA16	Have you needed to check your blood glucose levels more often during the breastfeeding period?	−0.358	−0.233	−0.385	−0.337	−0.138	1				
DIA17	Have you experienced more unstable blood glucose levels during the breastfeeding period?	−0.473	−0.225	−0.343	−0.361	0.009	**0.161**	1			
DIA18	Have you experienced more low blood glucose levels during the breastfeeding period?	−0.385	−0.197	−0.376	−0.364	−0.08	**0.253**	**0.341**	1		
DIA19	Have you received the support you needed from health care professionals in order to manage your diabetes during the breastfeeding period?	−0.061	−0.011	−0.035	−0.08	−0.124	−0.194	−0.177	−0.251	1	
DIA20	Have you needed support from family members to manage your diabetes during the breastfeeding period?	−0.233	−0.267	−0.207	−0.393	0.16	0.07	−0.017	−0.075	−0.01	1

Bold numbers indicate residual correlations higher than the relative cut off 0.09.
